# Developing genetic tools to exploit *Chaetomium thermophilum* for biochemical analyses of eukaryotic macromolecular assemblies

**DOI:** 10.1038/srep20937

**Published:** 2016-02-11

**Authors:** Nikola Kellner, Johannes Schwarz, Miriam Sturm, Javier Fernandez-Martinez, Sabine Griesel, Wenzhu Zhang, Brian T. Chait, Michael P. Rout, Ulrich Kück, Ed Hurt

**Affiliations:** 1 Biochemistry Center, University of Heidelberg, Heidelberg, Germany; 2 Laboratory of Cellular and Structural Biology and Laboratory of Mass Spectrometry and Gaseous Ion Chemistry, The Rockefeller University, New York, New York, USA; 3 Department for General and Molecular Botany, Ruhr-University Bochum, Bochum, Germany

## Abstract

We describe a method to genetically manipulate *Chaetomium thermophilum*, a eukaryotic thermophile, along with various biochemical applications. The transformation method depends on a thermostable endogenous selection marker operating at high temperatures combined with chromosomal integration of target genes. Our technique allows exploiting eukaryotic thermophiles as source for purifying thermostable native macromolecular complexes with an emphasis on the nuclear pore complex, holding great potential for applications in basic science and biotechnology.

The use of thermostable proteins from prokaryotic thermophiles (bacteria, archaea) has been extensively exploited in the past for various biotechnological applications and in structural biology (reviewed in^1^,^2^). In the eukaryotic kingdom, thermophily has been adopted in some fungi of which *Chaetomium thermophilum*, a thermophilic ascomycete species, can grow up to 60 °C with an optimum growth temperature around 50–55 °C. The thermostable nature of individual *C. thermophilum* proteins has previously been demonstrated and directly compared to their mesophilic homologues^3^,^4^. Moreover, with the recent release of a publicly available genome database with manually refined annotation^3^,^5^, *C. thermophilum* has served as a prosperous resource for thermostable eukaryotic proteins. Several proof-of-principle studies exploiting the *C. thermophilum* proteome for biotechnological applications^6^,^7^,^8^,^9^ or high-resolution 3D characterization of single proteins or reconstituted protein complexes^10^,^11^,^12^,^13^,^14^ have meanwhile been reported. However, these investigations required expression of *C. thermophilum* genes in heterologous mesophilic expression systems, as *C. thermophilum* has not yet been amenable for genetic manipulation. In an effort to fully exploit the potential of this thermophilic eukaryote as a source for biochemical and structural analyses of difficult proteins and derived complexes, we developed a transformation system for *C. thermophilum,* allowing the stable integration of constructs into the genome, which renders this thermophile accessible for affinity-purification of native thermostable proteins and protein complexes assembled under physiological conditions.

## Results and Discussion

**Figure 1:**
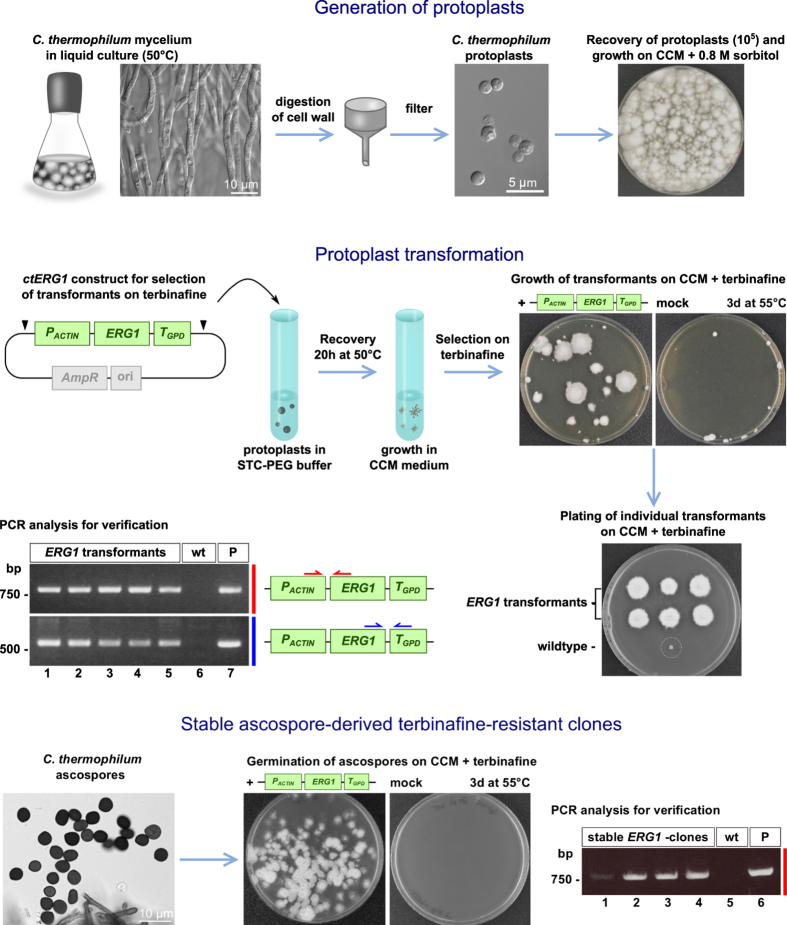
Schematic representation of the workflow for transformation of *C. thermophilum* protoplasts and selection of positive transformants. Protoplasts are generated from *C. thermophilum* mycelium growing in submerged cultures by digestion of fungal cell walls. Upon filtration, protoplasts are plated on osmotically stabilizing medium to assess viability. For transformation, protoplasts are mixed with the *P*_*ACTIN*_*-ERG1* plasmid for selection on terbinafine (see text for details). Arrowheads point to restriction sites for linearization. Upon recovery, mycelia are plated on solid media supplemented with terbinafine for selection of positive strains. Re-plating of individual transformed mycelia on selective medium confirms stable terbinafine resistance, whereas the wildtype does not grow on terbinafine. Left panel: PCR analysis with oligonucleotides specific for the *P*_*ACTIN*_*-ERG1* construct (depicted as red and blue arrows, respectively) performed on genomic DNA (gDNA) isolated from five independent *ERG1* transformants (lanes 1–5) to verify genomic integration of the construct. The expected products of 761 nt (upper panel) and 536 nt (lower panel), respectively, could be amplified, whereas no product was amplified from wildtype gDNA (wt). The *P*_*ACTIN*_*-ERG1* plasmid (P) was included as positive control (lane 7). Ascospores (bottom left) were generated from an *ERG1*-positive transformant to verify that the resistance trait remains stable. Upon plating of these ascospores on terbinafine, the *ERG1*- positive strain was able to germinate, whereas ascospores generated from the wildtype strain did not grow. Bottom right: PCR analysis performed on gDNA isolated from four independent ascospore-derived clones (lanes 1–4) verifies stable genomic integration of the *ERG1*-marker.

The genetic method developed in this study is based on polyethylene glycol (PEG) induced protoplast transformation that was successfully applied in the past for members of the *Pezizomycotina* clade (e.g. *Sordaria macrospora*^15^) to which *C. thermophilum* also belongs. For the generation of protoplasts from the thermophile, a mixture of different fungal cell wall degrading enzymes was applied to young mycelium of a wildtype *C. thermophilum* var. *thermophilum* (La Touche 1950; DSM-1495) growing as submerged cultures. The resulting protoplasts were collected by filtration and centrifugation, washed and subsequently subjected to the transformation procedure (Fig. 1) (see Methods for details).

The major challenge in establishing a transformation system for a eukaryotic thermophile that grows above 50 °C was the construction of a resistance marker that allows growth at this high temperature without denaturation, which is prerequisite to select for positive and stable transformants under continuous growth. A commonly applied dominant marker for effective selection of transformed fungal cells includes the hygromycin phosphotransferase (hph) from *Escherichia coli*, conferring resistance to hygromycin B^16^,^17^,^18^. Indeed, *Myceliophtora thermophila,* a milder thermophilic fungus, normally cultivated around 45 °C^19^, can be transformed using the *E. coli* hygromycin marker^20^. However, in the case of *C. thermophilum,* the hph marker could not withstand the high temperatures needed to cultivate *C. thermophilum* (N.K., unpublished results). Neither expression of a codon-optimized *hph* gene nor an engineered mutant version putatively conferring thermostability and constructed in analogy to a thermostable *hph* mutant isolated from the thermophilic archaeon *Sulfolobus solfataricus*^21^ led to any stable hygromycin-resistant transformants (data not shown). Thus, we assume that growth temperatures of 50–55 °C likely cause impaired enzyme activity or denaturation of the hph selection marker in *C. thermophilum*.

We therefore considered an alternative strategy, using a selection marker based on an entirely endogenous system derived from the thermophile. We selected the ergosterol biosynthesis pathway, because it is an important target for antimycotic drugs in fungi, as ergosterol is an essential component of fungal membranes^22^. Allylamines, like terbinafine, are capable of selectively repressing squalene epoxidase, a key enzyme of this pathway^23^,^24^, thus leading to impaired fungal growth in a vast variety of fungal species^25^. A common mechanism of resistance to such metabolic inhibitors can be mediated by overexpression of the drug target proteins^26^, as overexpression of the squalene epoxidase encoding gene *ergA* is able to confer terbinafine resistance in *Penicillium chrysogenum*^27^.

Accordingly, we cloned the *C. thermophilum* homologue of *ergA*, called *ctERG1*. In order to overexpress *ctERG1* from strong endogenous promoters, we further cloned the constitutively active promoter from the *actin* gene (*P*_*ACTIN*_) or the *trpC* (*P*_*TRPC*_) gene involved in tryptophan biosynthesis. Moreover, a transcription terminator fragment derived from the glyceraldehyde-3-phosphate dehydrogenase (*GPD*) gene was fused to the 3′ end of the gene construct, which allows for efficient PolII transcription termination (Fig. 1). Transformation and ectopic integration of these constructs into the *C. thermophilum* genome were expected to cause increased expression of the squalene epoxidase *ct*Erg1, thereby elevating the ergosterol level and circumventing the inhibitory effect of terbinafine. Following this strategy, we were able to obtain terbinafine resistant colonies (clones) on plates that were incubated at 50-55 °C for several days, routinely about 5–10 transformants per μg of linearized plasmid DNA (Fig. 1). Integration of the resistance marker into the genome was verified by PCR-based assays or Southern analysis (Fig. 1 and data not shown). As expected for ectopic integration of the selectable marker, the terbinafine resistance trait remained stable throughout multiple generations of cultivation on non-selective media as well as upon ascospore formation (Fig. 1).

**Figure 2:**
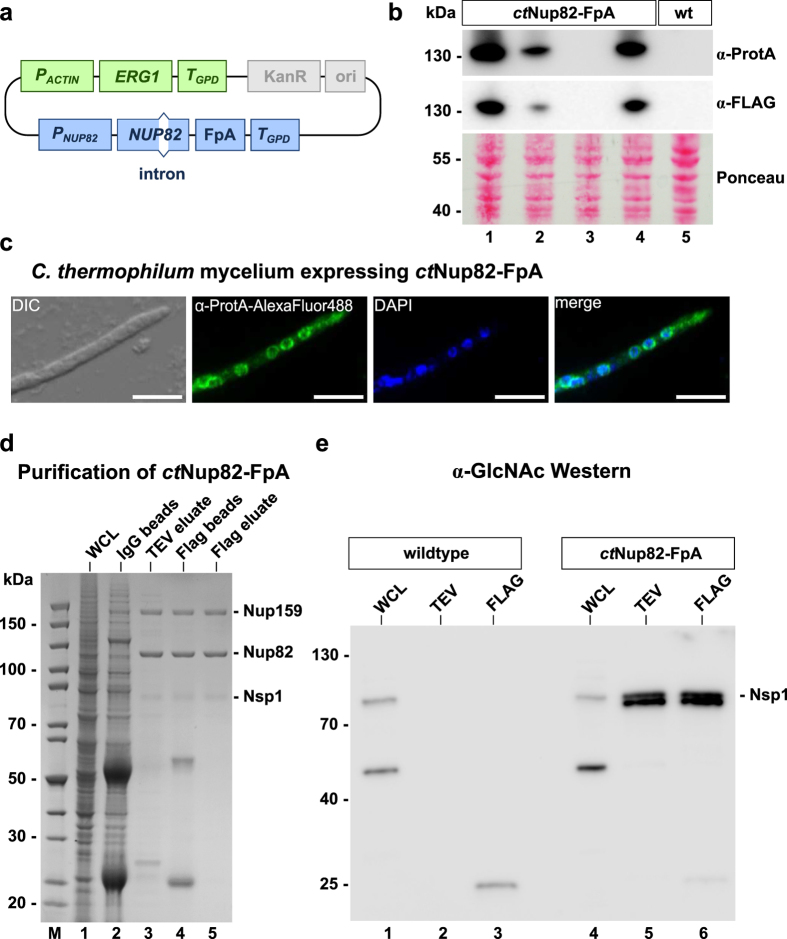
Design and expression of constructs for affinity purification of native *C. thermophilum* proteins. (**a**) Schematic depiction of the plasmid used for transformation and integration of a C-terminally ProtA-Flag tagged *ct*Nup82 protein. (**b**) Western analysis of four strains positive for the *P*_*ACTIN*_*-ERG1_P*_*NUP82*_*-NUP82-FpA* construct. Expression of the fusion protein was revealed with anti-ProteinA and anti-Flag antibodies on whole cell protein lysates, respectively, whereas no signal was detectable in the wildtype. Equal loading was confirmed by Ponceau S staining. A molecular weight standard with molecular weights (kDa) is depicted on the left. (**c**) Indirect immunofluorescence to detect *C. thermophilum* nuclear pore complexes. An antibody directed against Protein A in combination with an AlexaFluor488-conjugated secondary antibody decorates nuclear rims in the strain expressing *ct*Nup82-FpA, indicating that the fusion protein is functional and localizes to the NPC. DAPI staining was performed to visualize nuclei. DIC, GFP and DAPI channels as well as merged fluorescent images are shown. Scale bars – 10 μm. (**d**) Tandem-affinity purification of the native *ct*Nup82 complex. Samples are analyzed by SDS-PAGE and Coomassie staining. Whole cell lysate (WCL), the complex on IgG- and Flag-beads, respectively, the TEV- and final Flag-eluates are shown. Proteins were identified by mass-spectrometry. (**e**) Western analysis to identify O-linked glycosylation. Wildtype and *ct*Nup82-FpA expressing mycelium was subjected to affinity purification. WCL as well as TEV and Flag eluates, respectively, were probed with an antibody specific for O-linked N-acetylglucosamines (O-GlcNAc). This antibody detects a band with a molecular weight corresponding to *ct*Nsp1, both in the whole cell lysate and the purified trimeric complex, indicating O-GlcNAc modification of this nucleoporin. A molecular weight standard with molecular weights (kDa) is depicted on the left.

Subsequently, we aimed to transform the selectable *ERG1* marker together with gene constructs of interest, providing the possibility to affinity-purify authentic *C. thermophilum* proteins directly from the thermophile under physiological conditions. As proof of principle and to verify our method, we focused on proteins of the nuclear pore complex (NPC) that have been extensively studied in the yeast *Saccharomyces cerevisiae*, with a plethora of biochemical, structural and functional data being available^28^,^29^. Like in *C. thermophilum*^3^, the conserved NPC consists of about 30 different nucleoporins that are often organized in biochemically stable subcomplexes (modules), which can be affinity-purified using epitope-tagged Nups as bait (e.g.^28^,^30^). To test isolation of a native Nup82•Nup159•Nsp1 complex directly from *C. thermophilum*, we sought to tag the *ct*Nup82 subunit. A DNA fragment comprising the *NUP82* promoter and the *NUP82* ORF including its single intron was PCR-amplified from *C. thermophilum* genomic DNA. The use of genomic gene constructs including endogenous promoters and introns is expected to assure authentic gene expression levels close to physiological conditions. However, we have also tested the use of a stronger promoter (e.g. *P*_*ACTIN*_) fused to the target gene, which allows overexpression of the protein of interest (data not shown). Next, the *NUP82* ORF was C-terminally fused to a **FpA** cassette, consisting of DNA encoding the **F**lag-tag, the TEV **p**roteolytic cleavage site and the Prot**A** tag. The final plasmid containing the *P*_*ACTIN*_*:ERG1* selection marker and the *P*_*NUP82*_*:NUP82-FpA* construct (Fig. 2a) was linearized and transformed into *C. thermophilum* protoplasts, before terbinafine resistant colonies were selected. Ectopic integration of the construct was verified by PCR and Southern analysis (data not shown), whereas protein expression of *ct*Nup82-FpA was tested by preparing a whole cell lysate from the transformants, followed by SDS-PAGE and Western blotting using antibodies against the ProtA or Flag tag (Fig. 2b). In general, more than 50% of terbinafine resistant clones revealed expression of an FpA-carrying fusion protein of the expected size.

After identification of stable transformants expressing ProtA-Flag-tagged *ct*Nup82, we set out to establish a protocol for indirect immunofluorescence in *C. thermophilum* hyphae as a possible cell biology application. For this method, we used fixed mycelium of *ct*Nup82-FpA transformants that was mildly digested with lytic enzymes to permeabilize the cell wall (see Methods), before it was incubated with anti-ProtA antibodies followed by a second AlexaFluor488-conjugated antibody. Following this protocol, we observed a nuclear rim staining in *ct*Nup82-FpA expressing cells indicative of an NPC distribution, a pattern absent from the wildtype strain (Fig. 2c). This staining pattern was indistinguishable from one obtained by indirect immunofluorescence staining with an antibody directly raised against recombinantly produced *C. thermophilum* Nup82 (data not shown). This data demonstrates that ectopic expression of *ct*Nup82-FpA in *C. thermophilum* yielded functional nucleoporin *ct*Nup82 that was assembled into the NPC.

In the course of these studies, we also tested expression of the green fluorescent protein (GFP) either under control of the strong *P*_*ACTIN*_ promoter or C-terminally fused to *ct*Nup82 carrying its endogenous *P*_*NUP82*_ promoter. However, we could not detect any fluorescence signal, despite transformants being positively tested for integration of the *GFP* constructs (data not shown). Although this is negative data, it is possible that the optimal growth temperature of *C. thermophilum* (50–55 °C) is too high for either stable expression of GFP or developing its fluorophore, which is known to require correct and efficient protein folding^31^.

To prove that our technique allows not only expression but also affinity-purification of tagged bait proteins and co-enrichment of interacting factors assembled under physiological conditions, we affinity-purified *ct*Nup82-FpA from whole cell lysates under our standard buffer conditions, typically used for TAP-purification of complexes from yeast (see Methods for details). As shown by SDS-PAGE and Coomassie staining, *ct*Nup82-FpA significantly co-enriched two protein bands by tandem affinity-purification, which were identified by mass spectrometry to be *ct*Nup159 and *ct*Nsp1 (Fig. 2d). This data shows that ectopic expression of *ct*Nup82-FpA allowed isolation of the expected heterotrimeric Nup159•Nup82•Nsp1 complex^32^ directly from the thermophile without going through mesophilic expression systems, or *in vitro* reconstitution. Due to the ectopic integration of the *P*_*NUP82*_*:NUP82-FpA* construct, the respective endogenous wildtype *ctNUP82* allele was still present in the transformed strain. However, the tagged *ct* Nup82-FpA fusion protein efficiently competed with endogenous *ct*Nup82 for assembly, as revealed by efficient isolation of the native trimeric *ct*Nup82-complex and a distinct nuclear pore staining suggested by indirect immunofluorescence microscopy. Thus, for future studies using ectopic expression of tagged fusion proteins in *Chaetomium thermophilum*, it is important to ensure that the tagged fusion gene is functional, so that it can efficiently compete with the endogenous protein for assembly and/or function.

We noticed that the *ct*Nsp1 band co-purified with the *ct*Nup82 complex was less distinct than the other bands (Fig. 2d, lane 5), which could be due to post-translational protein modification. It is known that mammalian nucleoporins are post-translationally modified, e.g. by addition of O-linked N-acetylglucosamines (O-GlcNAc), and that Nup62, the human orthologue of *ct*Nsp1, is among the most O-GlcNAc-glycosylated proteins in a human cell^33^. Nup62 is modified by O-GlcNAc transferase (OGT), whose gene is not found in yeast, but present in the *C. thermophilum* genome. Hence, we tested whether *ct*Nsp1 carries this sugar modification by Western blotting using an antibody specific for O-GlcNAc. This analysis indicated that *ct*Nsp1 cross-reacts with this antibody, both in the whole cell lysate and the purified complex, suggesting O-GlcNAc modification of this nucleoporin occurs in the thermophile (Fig. 2e). Taken together, these data demonstrate that expression of *ct*Nup82-FpA in the authentic thermophile allows affinity-purification of a native nucleoporin subcomplex including post-translational modifications, which renders our method superior compared to heterologous expression systems.

**Figure 3:**
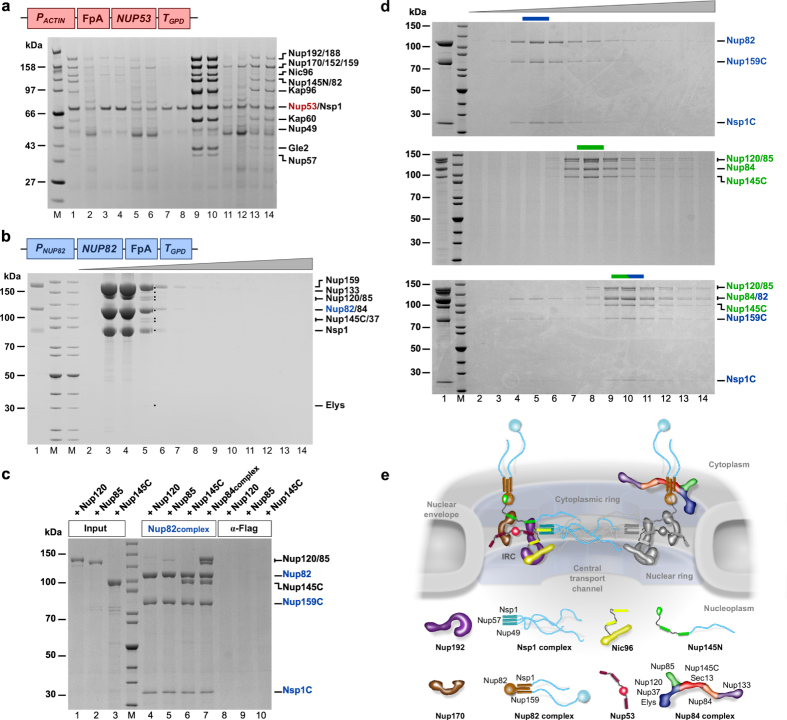
Biochemistry of native *C. thermophilum* nuclear pore subcomplexes and reconstitution of a Nup82-Nup84 super-complex. (**a**) Affinity capture of *ct*Nup53-FpA was performed on cleared lysates under various extraction conditions. Elution of the complexes was followed by SDS-PAGE and Coomassie staining. Proteins identified by mass spectrometric analysis from the complexes isolated in condition #9 are indicated. Extraction conditions (# 1–14) are presented in Supplementary Table 1. A molecular weight marker (M) is indicated on the left. (**b**) Sucrose gradient centrifugation of the *ct*Nup82 complex upon affinity capture of *ct*Nup82-FpA. Fractions were collected and analyzed by SDS-PAGE and Coomassie staining. Individual proteins were identified by mass-spectrometric analysis. (**c**) *In vitro* binding assay with an immobilized Nup82•Nup159C1•Nsp1C complex (*ct*Nup82 complex) and full length *ct*Nup120, *ct*Nup85 and *ct*Nup145C (*ct*Nup84 complex) (input, lanes 1–3). Samples are eluted via Flag-Peptide and analyzed by SDS-PAGE and Coomassie staining (lanes 5–11). MW: molecular weight, kDa: Kilodalton, α-Flag: Anti-Flag M2 Affinity Gel. (**d**) Glycerol-gradient centrifugation of *in vitro* reconstituted complexes. Fractions were collected and analyzed by SDS-PAGE and Coomassie staining. (top) *ct*Nup82 complex, (middle) *ct*Nup84 complex including *ct*Nup84, (bottom) *ct*Nup82-*ct*Nup84 complex including *ct*Nup84. (**e**) Schematic model for the connection of the Nup53-containing inner pore ring complex (IRC) with the Nup82 outer ring and the Nsp1 channel complex (left) and how the Nup82 complex might be linked to the Y-shaped octameric Nup84 complex within the NPC protomer.

To further elaborate on the biochemical possibilities to capture larger macromolecular complexes from the thermophile, we focused on *C. thermophilum* nucleoporin *ct* Nup53^3^, which was recently used as seed to reconstitute a huge nucleoporin super-complex composed of 11 subunits *in vitro*^32^. Hence, we wanted to test whether we can simplify this sophisticated and time-consuming reconstitution experiment by isolating this large assembly directly from the thermophile. Accordingly, we generated a *P*_*ACTIN*_*-FpA-NUP53-T*_*GPD*_ construct for ectopic expression and affinity-purification from *C. thermophilum.* We employed a recently developed screening method for optimizing affinity-purification of macromolecular complexes from whole cell lysates^34^. Applying this protocol for *C. thermophilum* mycelium expressing *ct*Nup53, we were able to enrich the entire NPC inner ring complex (composed of *ct*Nup192, *ct*Nup188, *ct*Nup170, *ct*Nic96 and *ct*Nup53) with the attached channel complex (*ct*Nsp1•*ct*Nup57•*ct*Nup49) and Nup82 subcomplex (*ct*Nup82•*ct*Nup159•*ct*Nsp1) as well as additional linker nucleoporins (*ct*Nup145N and *ct*Gle2), revealing an approximate stoichiometric ratio of the individual sub-units (Fig. 3a,e), which so far has not been achieved from mesophilic model organisms. Similarly, using *ct*Nup133-FpA, which is part of the octameric Y-complex^35^ as bait, we could isolate the endogenous *ct*Nup84-complex together with a number of other *ct*Nups, totally comprising more than half of all the *ct*Nups annotated for the thermophile^3^ (Supplementary Fig. 1).

Sucrose gradient ultracentrifugation of the *ct*Nup159•Nup82•Nsp1 complex purified from *C. thermophilum* revealed co-enrichment of a few bands, which were identified to be members of the *ct*Nup84 complex (Fig. 3b). This could point to a direct physical interaction between the Y-complex and the outer ring Nup82 complex. We sought to prove this unpredicted interaction by *in vitro* reconstitution. The isolated *ct*Nup82 complex (in this case derived from heterologous co-expression in yeast^32^) was incubated with the individual subunits of the Nup84 core complex, *ct*Nup120, *ct*Nup85 and *ct*Nup145C, that were also heterologously expressed in yeast. Apparently, only *ct*Nup145C was stoichiometrically bound to the *ct*Nup82 complex (Fig. 3c, lane 6), which in consequence facilitated the further recruitment of the other Y-complex subunits *ct*Nup120 and *ct*Nup85 (Fig. 3c, lane 7). This finding was verified by glycerol gradient centrifugation. Whereas the individual purified *ct*Nup82 and *ct*Nup84 complexes sedimented according to their molecular size in the corresponding fractions of the gradient, mixing of both complexes caused a clear shift towards the bottom of the gradient, where the *ct*Nup82-Nup84 super-complex sedimented (Fig. 3d). This data becomes relevant in the context of the cryo-tomographic structure of the human NPC scaffold, in which the Nup82 complex may come in close contact to the Y-complex^36^,^37^. Altogether, purification of native nucleoporin complexes from *C. thermophilum* gave novel insight into so far unrecognized physical interactions, which underscores the power of purifying the NPC and its modules from a eukaryotic thermophile.

In conclusion, we have developed genetic tools in *C. thermophilum*, which allow applications regarding affinity-purification of thermostable macromolecular complexes assembled under physiological conditions directly from the thermophile. This method holds great potential to obtain complicated protein complexes as well as RNPs from a thermophile, including native protein and RNA modifications. Moreover, the inherent thermostability of such assemblies will facilitate the identification of transient protein-protein interactions, which may escape during isolation of complexes from mesophiles. Thus, our method could pave the way for *C. thermophilum* to further develop into a model organism for biochemical and prospective structural analyses of eukaryotic macromolecular complexes, but also applications in biotechnology are expected to emerge.

## Methods

### Media and cultivation of *Chaetomium thermophilum*

Wildtype *Chaetomium thermophilum* (La Touche) *var. thermophilum* was obtained from DSMZ, Braunschweig, Germany (No. 1495). Mycelium was propagated on CCM medium (modified after^38^): 3 g sucrose, 0.5 g NaCl, 0.65 g K_2_HPO_4_∙3 H_2_O, 0.5 g MgSO_4_∙7H_2_O, 0.01 g Fe(III)sulfate-hydrate, 5 g tryptone, 1 g each of peptone and yeast extract and 15 g dextrin (potato) per liter, pH 7.0). For solid media, 20 g per liter agar was added. Plates were incubated at 52–55 °C. Submerged cultures were inoculated with mycelium scraped off a freshly grown agar plate and typically grown in 250 ml baffled Erlenmeyer flasks at 50 °C and 100 rpm in a rotary shaker for approximately 24 hours.

For large scale growth of mycelium, submerged cultures were shredded in a blender (4 × 20 sec) and used as inoculums for 2 L CCM in 5 L baffled Erlenmeyer flasks incubated at 50 °C and 90 rpm for 18 hours. For harvesting mycelium that was subsequently used for isolation of protein extracts or affinity-purification, the cultures were strained through a metal sieve (180 μm pore size), washed once with sterile water and residual liquid was removed with a vacuum filter. Cells were frozen in liquid nitrogen and subsequently ground to a fine powder in a cryogenic cell mill (Retsch MM400) in two cycles of five minutes each.

For the generation of ascospore-producing fruiting bodies, rice extract agar (supernatant of 75 g whole grain rice, boiled for two hours and supplemented with 15 g of agar per liter) was inoculated with shredded mycelium and incubated at 37 °C for approximately seven days. Spores were harvested through scraping the agar surface, filtrated through four layers of sterile gauze, washed in 1 M sorbitol and stored at −20 °C. Spores retained their ability to germinate on CCM agar for at least one year. For germination of ascospores on terbinafine, the minimal inhibitory concentration was determined and 0.1 μg/ml terbinafine was supplemented to solid CCM medium.

### DNA procedures, cloning of *C. thermophilum* nucleoporins for native affinity purification and recombinant expression in *S. cerevisiae* or *E. coli*

Molecular cloning techniques were based on protocols described in^39^. *E. coli* DH5α was used for plasmid propagation via standard procedures. Genomic DNA from *C. thermophilum* was isolated essentially as reported by^40^ and total RNA was isolated using the SV total RNA isolation Kit (Promega). cDNA was synthesized using Superscript-III Reverse Transcriptase (Invitrogen) and an Oligo d(T) primer according to the company’s instructions. cDNA was purified with the QIAquick PCR purification Kit (QIAGEN).

Construction of the terbinafine resistance marker was achieved by cloning the open reading frame (ORF) of *ctERG1* (CTHT_0032780). To obtain the *actin* promoter (*P*_*ACTIN*_) and the *trpC* promoter (*P*_*TRPC*_), a 1.5 kb fragment and a 1.2 kb fragment, respectively, upstream of the respective ORF (*actin* – CTHT_0062070; *trpC* – CTHT_0070860) was amplified from genomic DNA. *C. thermophilum* genes encoding nucleoporins were previously annotated^3^. For the generation and integration of affinity-tagged fusion constructs into the *C. thermophilum* genome, nucleoporin-encoding ORFs were PCR amplified from genomic DNA. For amplification of promoter regions, approximately 1.0–1.5 kb fragments upstream adjacent to the respective ORF were amplified. For termination of transcription, a 3′ transcription terminator DNA fragment (300 bp downstream of the glyceraldehyde-3-phosphate dehydrogenase ORF (*GPD;* CTHT_0004880) was fused to the ORF of interest. All plasmids used for transformation into *C. thermophilum* protoplasts are listed in Supplementary Table 2.

To clone *C. thermophilum* nucleoporins for recombinant expression, the respective gene was PCR amplified from either cDNA or genomic DNA, when no intron was present, and inserted into appropriate expression plasmids. All constructs used in this study are listed in Supplementary Table 3.

### Transformation of *C. thermophilum* and expression tests

Protoplasts were generated using a submerged culture of the *C. thermophilum* wildtype strain, shredded in a kitchen blender (4 × 20 sec), as inoculum for 300 ml CCM medium, which was incubated at 50 °C and 90 rpm for approximately 20 hours. The mycelium was strained through a metal sieve, washed twice with PP buffer (0.8 M sorbitol, 0.013 M Na_2_HPO_4_, 0.045 M KH_2_PO_4_, pH 6.5), subsequently subjected to 100 ml Erlenmeyer flasks containing 40 ml digestion solution (30 mg/ml lysing enzymes from *Trichoderma harzianum* (Sigma Aldrich cat. no. L1412) and 10 mg BSA fraction V in PP buffer) and incubated at 30 °C and 110 rpm for three hours. The resulting protoplasts were filtered through a funnel (pore size 1) and collected via centrifugation (2400 rpm, 8 min, 4 °C), followed by two washing steps in PP buffer and one washing step in STC buffer (0.8 M sorbitol, 80 mM CaCl_2_, 10 mM Tris-HCl, pH 7.5). The amount of protoplasts was determined in a hemocytometer and from a standard 300 ml culture, we routinely obtained about 1–2 × 10^10^ protoplasts, which were subsequently adjusted to 1 × 10^8^–5 × 10^8^ per ml in STC buffer. For the determination of the recovery rate, different amounts of protoplasts were plated on CCM medium supplemented with 0.8 M sorbitol, incubated at 50 °C for one day and the resulting colonies were counted. Generally, a recovery rate of 5–10% was determined. For transformation of protoplasts with plasmid DNA, 200 μl of the protoplast solution was mixed with 2.5 μl heparin (10 mg/ml), 2 μl spermidine trihydrochloride (50 mM), 1 μl aurintricarboxylic acid (0.4 M) as nuclease inhibitor, 40 μl STC/PEG solution (40% PEG_6000_ (w/vol) in STC buffer) and 5–10 μg of linearized plasmid DNA and incubated on ice for 20 min. Then, 750 μl of STC/PEG solution was added prior to incubation at room temperature for 10 min to allow the transformation to proceed. The protoplasts were again collected via centrifugation and recovered in 3 ml CCM medium supplemented with 0.8 M sorbitol at 50 °C and 600 rpm for approximately 20 hours. After recovery, the cells were plated on CCM agar plates supplemented with 0.8 M sorbitol as well as terbinafine hydrochloride (Sigma Aldrich) at a final concentration of 0.5 μg/ml for selection and incubated at 50–55 °C for two to three days. Expression of affinity-tagged fusion proteins was tested via immunoblots of whole cell protein lysates obtained from 100–150 mg of mycelium, ground in 1 ml NB-Hepes buffer (20 mM Hepes, pH 7.5, 150 mM NaCl, 50 mM K(OAc), 2 mM Mg(OAc)_2_, 1 mM dithiothreitol, 5% glycerol, and 0.1% (vol/vol) IGEPAL^®^ CA-630) and 500 μl of zirconia beads (Ø 0.5 mm, Carl Roth) in a Mini Bead Beater (5000 rpm at 4 °C, 2 × 20 sec, 4 runs). The lysates were cleared via centrifugation (14.000 rpm at 4 °C for 20 min) and supernatants were analyzed by SDS-PAGE and Western blotting, performed using PAP (Sigma Aldrich cat. no. P1291) and/or Flag^®^ (Sigma Aldrich cat. no. A8592) antibodies according to the manufacturer’s instructions.

### Affinity-purification of native protein complexes from *C. thermophilum*

Ground frozen mycelium was thawed on ice in NB-Hepes buffer containing 0.1% (vol/vol) IGEPAL^®^ CA-630 or 1% (vol/vol) Triton^TM^ X-100, including SIGMA*FAST* complete protease inhibitor cocktail (Sigma Aldrich) at a ratio of approximately 1 ml buffer per gram of cells. The lysate was cleared (20.000 rpm at 4 °C for 30 min) and the Flag-TEV-ProteinA (FpA) tagged proteins were affinity-purified from the supernatant using 300 μl IgG-Sepharose suspension (IgG-Sepharose 6 Fast Flow; GE Healthcare). Upon washing, proteins were eluted by TEV protease in a 2.5 ml Mobicol column (MoBiTec) at 16 °C for two hours and subsequently incubated with 50 μl ANTI-Flag^®^ M2 affinity gel (Sigma Aldrich) at 4 °C for one hour. Bound proteins were eluted by use of 100 μg/ml Flag peptide (Sigma Aldrich) at 4 °C for 30–60 min. Eluates were separated by SDS-PAGE and analyzed via Colloidal Coomassie staining and mass spectrometry. For the detection of glycosylation of *ct*Nsp1, an O-linked N-acetylglucosamine antibody (abcam cat. no. ab2739, 1:1000 in PBS + 0.05% Tween-20/5% milk) was applied. Sucrose gradient ultra-centrifugation was performed with the Flag eluates, which were loaded onto a continuous sucrose gradient (10–30% (w/vol) in NB-Hepes buffer), centrifuged at 26.000 rpm for 16 hours in a SW60 Ti swinging bucket rotor (Beckman Coulter) and fractionated.

To test different solvent compositions for *ct*Nup53 and *ct*Nup133, cryo-milled mycelium was dispensed on a 24-well format microtiter plate and subjected to a magnetic-bead based affinity capture procedure according to the protocol published by Hakhverdyan *et al*.^34^. The formulations of extractants used for the experiment are presented in Supplementary Table 1. Mass spectrometric identification of proteins was carried out as described^34^.

### Immunostaining of *C. thermophilum* nucleoporins and fluorescence microscopy

Immunostaining was adapted from the protocol published by Zekert and Fischer^41^. In brief, mycelium from an overnight culture was collected in a metal sieve and washed once with phosphate buffered saline (PBS). The edges of the mycelia were cut in small pieces, streaked on polytetrafluorethylene (PTFE)-coated microscope slides and air-dried. Cells were fixed for 30 min with formaldehyde and digested for one hour in digestion solution (50 mg/ml lysing enzymes from *Trichoderma harzianum*) in 50 mM sodium citrate, pH 5.8, with 50% egg white), washed three times in PBS, incubated in pre-cooled methanol at −20 °C for 10 min and blocked with PBS supplemented with 5% milk powder (w/vol) prior to incubation with the first antibody (α-ProteinA; Sigma Aldrich cat. no. P3775) in a 1:2000–1:5000 dilution in PBS/5% milk overnight at 4 °C, followed by three washes in PBS with 0.05% Tween-20. Alternatively, an antibody raised in rabbit against recombinant *C. thermophilum ct*Nup82 was used in a 1:500 dilution in PBS/5% milk. Prior to use, the antibody was affinity-purified from rabbit serum according to^42^. As secondary antibody, an AlexaFluor488-conjugated α-rabbit antibody (life technologies cat. no. A11008, 1:500 in PBS/5% milk) was used. Upon incubation at 4 °C for one hour and three washes in PBS with 0.05% Tween-20, cells were subjected to 4,6-diamidino-2-phenylindole (DAPI) staining by incubation in DAPI-staining solution (100 ng/ml in 60 mM Tris-HCl, pH 7.2, 200 mM NaCl, 1.6% BSA) for 10 min at room temperature followed by three washes in PBS. Then cover slips were mounted on the microscope slides in a drop of Mowiol and the cells were stored in the dark or directly subjected to fluorescence microscopy using an Imager Z1 microscope (Carl Zeiss) equipped with a 100x/63x NA 1.4 Plan-Apochromat oil immersion objective lens. Standard filter sets for DAPI and GFP were used for epifluorescence analysis. Images were acquired with an AxioCam MRm camera (Carl Zeiss). All image processing including adjustment of brightness, contrast and gamma-values was performed using AxioVision 4.8.2.0 software (Carl Zeiss).

### Expression of recombinant *C. thermophilum* nucleoporins in yeast and *E. coli*

For recombinant expression of individual nucleoporins from *C. thermophilum*, plasmids harboring the nucleoporin-encoding gene of interest under control of the *ADH1* or *GAL1/GAL1-10* promoter, respectively, were transformed into *S. cerevisiae* strain DS1-2b^43^. To perform simultaneous co-expression of multiple genes, the coding sequences were inserted into appropriate yeast expression vectors, as described previously^35^. For expression of constructs under control of the constitutive *ADH1* promoter, cells were grown under continuous agitation in SDC dropout media at 30 °C to an OD_600_ _nm_ of 4 and subsequently harvested. Strains harboring constructs with the inducible *GAL1/GAL1-10* promoter were grown in SRC dropout media at 30 °C to an OD_600_ _nm_ of 4. At this point, media were supplemented with 2-fold concentrated YPG medium in a 1:1 ratio to induce expression. Cultures were grown for additional 5 hours prior to harvesting the cells. For bacterial expression, plasmids with the *lacI* repressed T7 promoter were transformed into *E. coli* strain BL21^+^. Cells were grown in selective LB media at 37 °C until an OD_600_ _nm_ of 0.5. At that point 0.7 mM IPTG (Isopropyl-β-D-thiogalactopyranosid) was supplemented, the cells were further grown at 23 °C for three hours and harvested.

### Purification of recombinant *C. thermophilum* nucleoporins, reconstitution of subcomplexes and *in vitro* binding assays

Cell lysis was performed by cryogenic grinding in a cell mill (Retsch MM400). Lysed cells were resuspended in NB-Hepes buffer containing SIGMA*FAST* complete protease inhibitor cocktail and 0.1% (vol/vol) IGEPAL^®^ CA-630, and the lysate was cleared by centrifugation. For purification, lysates were incubated at 4 °C for 60 min with corresponding affinity beads: ProtA-TEV tagged proteins were purified using IgG-Sepharose 6 Fast Flow (GE Healthcare), GST-TEV tagged proteins using Protino Glutathion Agarose 4B (GSH Agarose, Macherey-Nagel), His6 tagged proteins with His-Select Nickel affinity gel (Ni-Gel, Sigma Aldrich) and Flag tagged proteins using ANTI-Flag^®^ M2 affinity gel (Sigma Aldrich). After binding, affinity beads were washed extensively using NB-Hepes-W buffer (NB-Hepes containing 0.01% (vol/vol) IGEPAL^®^ CA-630). Elution of IgG and GSH affinity bound proteins was achieved by cleavage with the TEV protease for 60 min at 16 °C in NB-Hepes-W buffer containing 1 mM dithiothreitol. Ni-Gel bound protein was eluted using NB-Hepes-W buffer containing 300 mM imidazole. For elution of Flag bound protein, the affinity gel was incubated with NB-Hepes-W containing 100 μg/ml Flag^®^ peptide (Sigma Aldrich) for 30 min at 4 °C.

The reconstitution of the heterotrimeric *ct*Nup82 complex from *C. thermophilum*, consisting of *ct*Nup82, *ct*Nup159C and *ct*Nsp1C, was performed as described^32^. Briefly, the three constructs were co-expressed in *S. cerevisiae* and a split-tag purification of the complex was performed. In the first step, the *ct*Nup82 complex was immobilized to Ni-Gel via *ct*Nsp1C:His6, eluted with imidazole and further purified in a second step via Flag tagged *ct*Nup159C. The purified *ct*Nup82 complex was used as bait in the following binding experiments. As prey, individual *ct*Nup84 complex nucleoporins were purified and eluted as outlined above. Alternatively, the heterotrimeric *ct*Nup84 complex, consisting of *ct*Nup120, *ct*Nup85 and *ct*Nup145C, was co-expressed in *S. cerevisiae*, purified via ProtA-TEV tagged *ct*Nup85 and eluted with TEV protease as illustrated. For binding experiments or reconstitution of higher-order complexes, the bait protein complex was incubated with 10-fold excess of prey protein for 60 min at 16 °C. After extensive washing, the bait proteins were eluted as described above and analyzed by SDS-PAGE and Coomassie staining.

For gradient ultra-centrifugation, the purified and reconstituted heterotrimeric *ct*Nup82 complex and the heteroheptameric *ct*Nup82-Nup84 super-complex, respectively, was loaded onto continuous glycerol gradient (10–30% w/vol in NB-Hepes buffer) and centrifuged at 33.000 rpm for 16 hours in a SW60 Ti swinging bucket rotor (Beckman Coulter). 200 μl fractions were collected and analyzed by SDS-PAGE and Coomassie staining.

## Additional Information

**How to cite this article**: Kellner, N. *et al*. Developing genetic tools to exploit *Chaetomium thermophilum* for biochemical analyses of eukaryotic macromolecular assemblies. *Sci. Rep.* **6**, 20937; doi: 10.1038/srep20937 (2016).

## Supplementary Material

Supplementary Information
