# Supplementary material for: Developing genetic tools to exploit Chaetomium thermophilum for biochemical analyses of eukaryotic macromolecular assemblies
Source: Sci Rep. 2016 Feb 11;6:20937. doi: 10.1038/srep20937 (PMC4750058; doi:10.1038/srep20937)
Supplement: Supplementary Information [file srep20937-s1.pdf]

Supplementary Information

Developing genetic tools to exploit *Chaetomium thermophilum* for biochemical analyses of eukaryotic macromolecular assemblies

Nikola Kellner[1], Johannes Schwarz[1], Miriam Sturm[1], Javier Fernandez-Martinez[2], Sabine Griesel[1], Wenzhu Zhang[2], Brian T. Chait[2], Michael P. Rout[2], Ulrich Kück[3] & Ed Hurt[1]

[1]Biochemistry Center, University of Heidelberg, Heidelberg, Germany

[2]Laboratory of Cellular and Structural Biology and Laboratory of Mass Spectrometry and Gaseous Ion Chemistry, The Rockefeller University, New York, New York, US

[3]Department for General and Molecular Botany, Ruhr-University Bochum, Bochum, Germany

Hurt_Supplementary Figure 1

Affinity purification of native *ct*Nup133-FpA under different buffer conditions

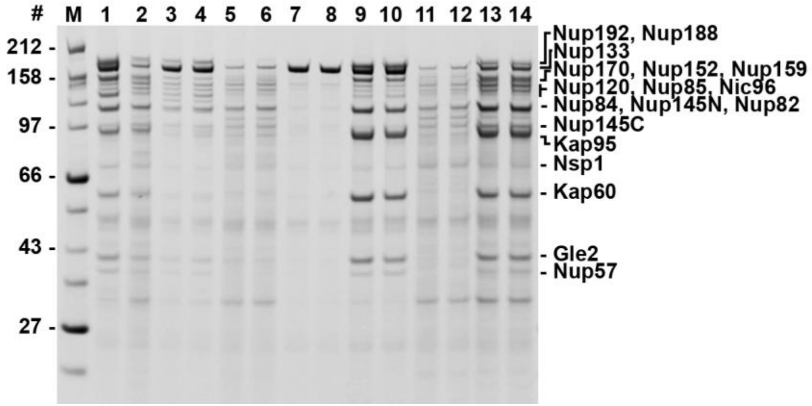

Supplementary Figure 1 Biochemistry of native *C. thermophilum* nuclear pore subcomplexes. Affinity capture of *ct*Nup133-FpA was performed on cleared lysates under various extraction conditions. Elution of the complexes was followed by SDS-PAGE and Coomassie staining. Proteins identified by mass spectrometric analysis from the complexes isolated in condition #9 are indicated. Extraction conditions (# 1-14) are presented in Supplementary Table 1. A molecular weight marker (M) is indicated on the left.

Supplementary Table 1: Extraction solvents used in affinity capture screens

Condition #	Components
1	1.5M Ammonium acetate pH 7.0, 0.5% Triton
2	1.5M Ammonium acetate pH 7.0, 0.1% CHAPS
3	125m Sodium phosphate pH 7.0, 0.5% Triton
4	250m Sodium phosphate pH 7.0, 0.5% Triton
5	125m Sodium phosphate pH 7.0, 0.1% CHAPS
6	250m Sodium phosphate pH 7.0, 0.1% CHAPS
7	40mM Tris pH 8.0, 50mM sodium citrate, 2mM EDTA, 125mM NaCl, 0.5% Triton
8	40mM Tris pH 8.0, 50mM sodium citrate, 2mM EDTA, 250mM NaCl, 0.5% Triton
9	40mM Tris pH 8.0, 250mM sodium citrate, 2mM EDTA, 250mM NaCl, 0.5% Triton
10	40mM Tris pH 8.0, 250mM sodium citrate, 2mM EDTA, 500mM NaCl, 0.5% Triton
11	40mM Tris pH 8.0, 50mM sodium citrate, 2mM EDTA, 125mM NaCl, 0.1% CHAPS
12	40mM Tris pH 8.0, 50mM sodium citrate, 2mM EDTA, 250mM NaCl ,0.1% CHAPS
13	40mM Tris pH 8.0, 250mM sodium citrate, 2mM EDTA, 250mM NaCl, 0.1% CHAPS
14	40mM Tris pH 8.0, 250mM sodium citrate, 2mM EDTA, 500mM NaCl, 0.1% CHAPS

Supplementary Table 2: Plasmid constructs for transformation of *C. thermophilum* used in this study

Plasmid	Relevant information	Reference
P_{ACTIN}*:ctERG1:*T_{GPD}	ColE1 origin, LEU2, AmpR	This study
P_{TRPC}*:ctERG1:*T_{GPD}	ColE1 origin, LEU2, AmpR	This study
P_{ACTIN}*:ctNUP82:Flag-TEV-ProtA:*T_{GDP}	pRSFduet origin, KanR, P_{ACTIN}*:ctERG1:*T_{GPD}	This study
P_{NUP82}*:ctNUP82:Flag-TEV-ProtA:*T_{GPD}	pRSFduet origin, KanR, P_{ACTIN}*:ctERG1:*T_{GPD}	This study
P_{ACTIN}*:ProtA-TEV-Flag:ctNup53:*T_{GPD}	pRSFduet origin, KanR, P_{ACTIN}*:ctERG1:*T_{GPD}	This study
P_{ACTIN}*:ProtA-TEV-Flag:ctNup133:*T_{GPD}	pRSFduet. origin, KanR, P_{ACTIN}*:ctERG1:*T_{GPD}	This study

Supplementary Table 3: Plasmid constructs for *in vitro* reconstitution of *C. thermophilum* NPC subcomplexes used in this study

Plasmid	Relevant information	Reference
YePlac112 ProtA-TEV_ctNup120	2µ, pGAL1-10, TRP1, LEU2D, AmpR	This study
pADH181 ProtA-TEV_ctNup85	2µ, pADH1, LEU2, AmpR	1
pADH181 ProtA-TEV_ctNup145C	2µ, pADH1, LEU2, AmpR	1
YePlac112 ctNup145C(P2)	2µ, pGAL1-10, TRP1, AmpR	1
pADH181 ProtA-TEV_ctNup84	2µ, pADH1, LEU2, AmpR	This study
YePlac112(L2D) ProtA-TEV_ctNup133	2µ, pGAL1-10, TRP1, LEU2D, AmpR	This study
YePlac181 ctNup120(P2) ProtA-TEV_ctNup85(P1)	2µ, pGAL1-10, LEU2, AmpR	1
YePlac195 ctNup82	2µ, pGAL1-10, URA3, AmpR	2
YePlac181 Flag_ctNup159C1	2µ, pGAL1-10, LEU2, AmpR	This study
YePlac112 ctNsp1C_His6	2µ, pGAL1-10, TRP1, AmpR	2
YePlac112 ctNup82_TEV-ProtA	2µ, pGAL1-10, TRP1, AmpR	This study
pET24D GST-TEV_ctNsp1C	f1 origin, T7 promoter, pBR322 origin, KanR2	This study
pET24D GST-TEV_ctNup159C1_His6	f1 origin, T7 promoter, pBR322 origin, KanR2	This study

1. Thierbach, K. et al. Protein interfaces of the conserved Nup84 complex from *Chaetomium thermophilum* shown by crosslinking mass spectrometry and electron microscopy. *Structure* **21**, 1672-1682 (2013).
2. Fischer, J., Teimer, R., Amlacher, S., Kunze, R. & Hurt, E. Linker Nups connect the nuclear pore complex inner ring with the outer ring and transport channel. *Nature structural & molecular biology* (2015).
